# Immune Cell Regulation of White Adipose Progenitor Cell Fate

**DOI:** 10.3389/fendo.2022.859044

**Published:** 2022-03-29

**Authors:** Irem Altun, Xiaocheng Yan, Siegfried Ussar

**Affiliations:** ^1^ Research Group Adipocytes and Metabolism, Institute for Diabetes and Obesity, Helmholtz Diabetes Center, Helmholtz Zentrum München, German Research Center for Environmental Health GmbH, Neuherberg, Germany; ^2^ German Center for Diabetes Research (DZD), Neuherberg, Germany; ^3^ Department of Medicine, Technische Universität München, Munich, Germany

**Keywords:** preadipocyte, immune cells, proliferation, differentiation, adipose tissue expansion, obesity, adipose precursor cells

## Abstract

Adipose tissue is essential for energy storage and endocrine regulation of metabolism. Imbalance in energy intake and expenditure result in obesity causing adipose tissue dysfunction. This alters cellular composition of the stromal cell populations and their function. Moreover, the individual cellular composition of each adipose tissue depot, regulated by environmental factors and genetics, determines the ability of the depots to expand and maintain its endocrine and storage function. Thus, stromal cells modulate adipocyte function and vice versa. In this mini-review we discuss heterogeneity in terms of composition and fate of adipose progenitor subtypes and their interactions with and regulation by different immune cell populations. Immune cells are the most diverse cell populations in adipose tissue and play essential roles in regulating adipose tissue function *via* interaction with adipocytes but also with adipocyte progenitors. We specifically discuss the role of macrophages, mast cells, innate lymphoid cells and T cells in the regulation of adipocyte progenitor proliferation, differentiation and lineage commitment. Understanding the factors and cellular interactions regulating preadipocyte expansion and fate decision will allow the identification of novel mechanisms and therapeutic strategies to promote healthy adipose tissue expansion without systemic metabolic impairment.

## Introduction

White adipose tissue (WAT) is an important endocrine organ playing a key role in energy homeostasis and insulin action (1–4). Therefore, its development and expansion is crucial to establish and maintain a functional metabolism. However, excessive fat accumulation results in obesity, predisposing most people to the development of insulin resistance and the metabolic syndrome. A gain in fat mass results in various changes in adipose depots including remodeling of the extracellular matrix (ECM), recruitment and activation of immune cells, upregulation of pro-inflammatory cytokines and changes in adipogenesis and adipocyte function (5). However, a number of studies described a metabolically healthy obese (MHO) phenotype (6–9). One characteristic of these subjects is that in contrast to metabolically unhealthy obesity (MUO), these individuals accumulate fat preferentially in the subcutaneous rather than visceral depots. Despite their adiposity MHO individuals are insulin sensitive, glucose tolerant and do not display ectopic lipid accumulation in organs such as the liver or skeletal muscle (7, 8). The underlying mechanisms and temporal stability of healthy adipose tissue expansion are still incompletely understood. However, it appears that there are principle mechanisms available allowing the metabolically safe storage of very large quantities of energy in adipose tissue without impairing adipose or systemic metabolic function. Various genetic polymorphisms, altering adipocyte function, could contribute to the MHO phenotype (10, 11). Differences in the preadipocyte pool, its size, composition and proliferative capacity as well as the interaction with other cells of the stromal compartment, especially the diverse immune cell populations could also play an important role in the MHO phenotype. However, these interactions in regulation of healthy adipose tissue expansion are less understood. In addition to white adipose tissue function, differences in brown fat function could also play a role in MHO. Brown adipocytes, in contrast to white adipocytes, do not act as site of energy storage, but rather dissipate energy to produce heat through UCP-1 mediated mitochondrial uncoupling (12, 13). Others and we have identified heterogeneity within murine brown adipocyte progenitors and brown adipocytes modulating overall brown fat activity and systemic metabolism (14, 15). Thus, inter-individual differences in brown adipocyte composition could also modulate the physiological response to weight gain. Moreover, the role of beige adipocytes, which refers to brown adipocyte-like cells within white adipose tissue depots, has also not been extensively studied in the context of MHO. However, in this review we will focus on factors and cell populations that regulate the fate of white adipose progenitor cells (APCs), preadipocytes and tissue homeostasis with a main focus on immune cells.

## Adipose Tissue During Development and Obesity

In humans, white adipose tissue starts to develop around 14 weeks of gestation as mesenchymal lobules that differentiate into primitive fat lobules. This is initiated by mesenchymal cell condensation resulting in the development of a capillary network and vascularization, followed by the proliferation of preadipocytes and formation of definitive fat lobules, which can be observed before week 28. The number of fat lobules is determined by week 23, while the size continuous to increase until week 29 (16, 17). In mice, subcutaneous white adipose tissue (sWAT) develops prenatally, starting between E14 and E18, while visceral white adipose tissue (vWAT) develops starting at P1 (18). In humans, the total number of adipocytes increases during childhood and adolescence, reaching a constant number at around 20 years of age, irrespective of the overall fat mass stored in a person. This indicates that inter-individual differences in adipocyte numbers are defined early in life and not by weight gain in adulthood (19, 20). However, obesity can also increase the estimated number of adipocytes, albeit it remains unclear how the subjects in this study differ from those in other studies that have not observed this increase in adipocyte numbers in adulthood (21).

Regardless, turnover of adipocytes and progenitor proliferation appear much more dynamic. In mice, around 5% of preadipocytes replicate and 1-5% of adipocytes are replaced per day. In humans the annual adipocyte turnover is around 10% (19, 22). Thus, adipocyte death, *de novo* differentiation and proliferation of adipocyte progenitors need to be tightly regulated to maintain a constant adipocyte number throughout life. Moreover, species differences in turnover of adipocytes and adipocyte progenitor proliferation should be considered when translating murine research data to humans. This is especially true for any therapeutic strategies aiming to increase the number of thermogenic beige adipocytes in WAT through *de novo* differentiation or promoting hyperplasia over hypertrophy.

In addition, obese individuals generate a higher number of new adipocytes every year compared to lean individuals. Conversely, significant body fat loss can also induce proliferation of adipocyte progenitor cells, as this was observed following bariatric surgery (19, 21). Thus, both weight gain and loss can have effects on the adipocyte progenitor pool. In mice, two weeks of high fat diet (HFD) feeding increased the proliferation of preadipocytes in visceral adipose tissue either compared to mice fed a chow diet (CD) or switched to a CD from a HFD (23). This effect was lost after 7 weeks of HFD feeding. However, prolonged HFD feeding increased the number of *de novo* differentiated adipocytes. Thus, there are complex interactions between a hypercaloric state requiring additional storage of energy in fat, progenitor expansion, and adipose tissue growth by either increasing the number of adipocytes (hyperplasia) or increasing adipocyte size (hypertrophy) (19, 24).

Expansion of adipose tissue through an increase in adipocyte number requires regulated proliferation of the adipocyte progenitor population to prevent progenitor exhaustion enabling continued adipogenesis throughout life. Thus, a variety of cell types, such as immune, endothelial and nerve cells interact with different subpopulations of APCs and preadipocytes to regulate adipose tissue function and growth, as well as homeostasis of the preadipocyte pool.

## Heterogeneity and Hierarchy in Adipose Progenitor Cells

The definition of what exactly is an adipocyte progenitor cell is often unclear. As previously discussed, various cell types, such as mesenchymal stem cell, pericytes, endothelial cells and others can give rise to adipocytes (5). Moreover, advances in genetic lineage tracing and single cell technologies have identified intermediate states and distinct adipocyte progenitor lineages, giving rise to distinct adipocyte subpopulations (25–27). Following the definitions suggested by Sakers et al., we use the term preadipocytes to refer to cells with the general ability to differentiate into adipocytes (25). In addition, we distinguish, where possible, APCs that have a higher proliferative capacity, stem like properties and are able to maintain the progenitor niche in the tissue. In the following sections, we use the more general term preadipocytes whenever it is unclear if APCs or preadipocytes were studied.

**Figure 1:**
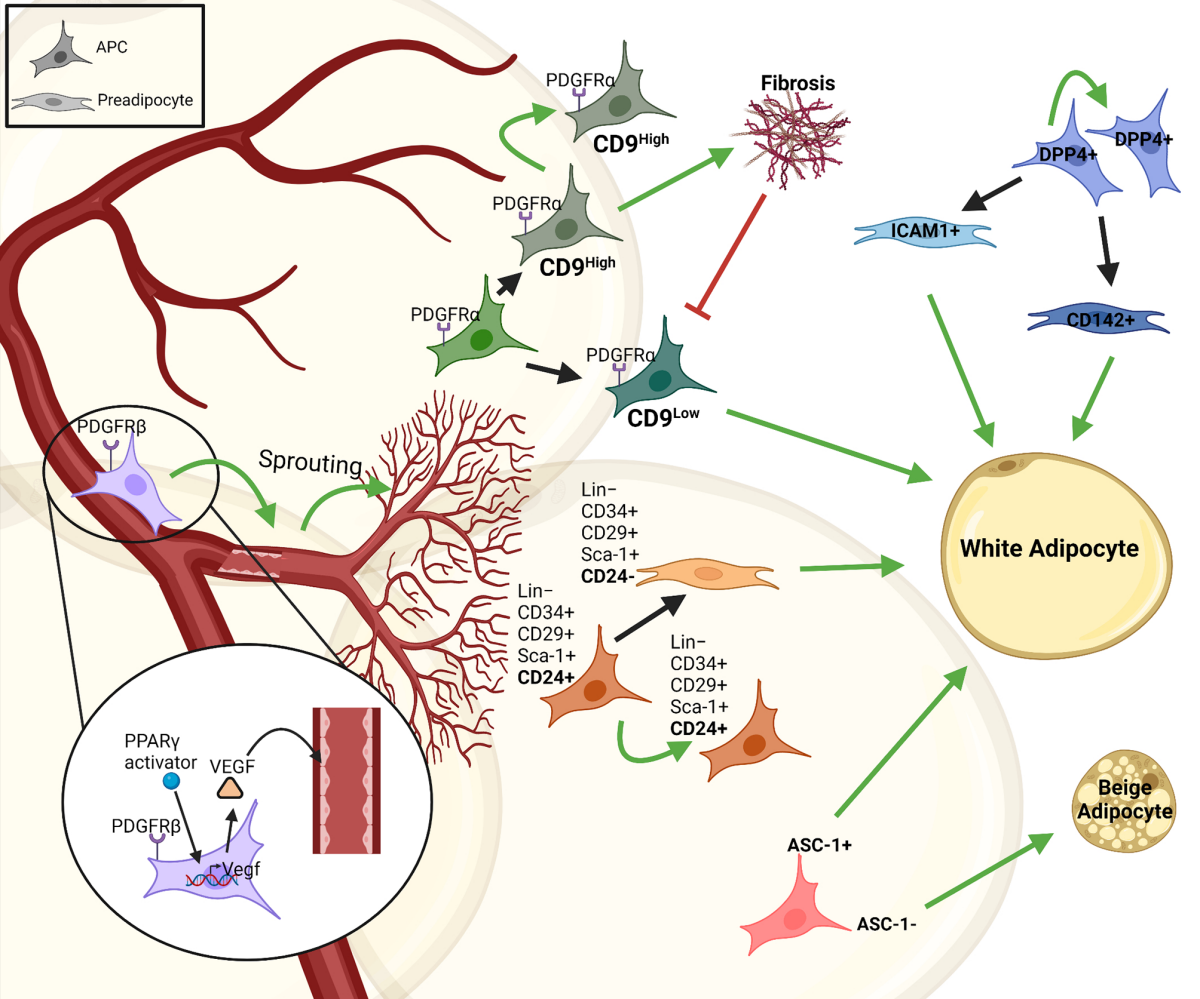
Heterogeneity and interactions of adipose progenitor cells. There are different subtypes of APCs with self-renewal properties in adipose tissue that give rise to various subpopulations of committed preadipocytes (e.g. CD24^-^, ICAM1^+^ or CD142^+^ cells). This proliferative/self-renewal capacity is represented by a green arrow that give rise to the same cell type. Some of these progenitors generate preadipocytes that differentiate into white adipocytes, such as; ASC1^+^, CD24^+^, DPP4^+^, while some are responsible for vascular-APC interaction including PDGFRβ^+^PPARγ^+^, others contribute to fibrosis in the tissue like PDGFRα^+^CD9^high^ cells and some like ASC-1^-^ cells give rise to beige adipocytes. The figure was generated using BioRender.com.

Analysis of the stromal vascular fraction (SVF) from WAT identified adipocyte progenitor populations that are Lin^−^:CD34^+^:CD29^+^:Sca-1^+^:CD24^+^ (CD24^+^) or Lin^−^:CD34^+^:CD29^+^:Sca-1^+^:CD24^−^ (CD24^-^), which was confirmed using PDGFRα-cre dependent reporter mice. Around 53% of the stromal vascular cells are CD24^-^ preadipocytes, while only 0.08% were CD24^+^ APCs. These studies established a principle hierarchy where CD24 expressing APCs give rise to committed CD24^-^ preadipocytes (**Figure 1**) (28, 29). Moreover, transplantation of CD24^+^ APCs but not CD24^-^ preadipocytes into A-zip lipodystrophic mice were able to form a complete functional WAT, demonstrating the role of APCs in regulating stromal interactions and tissue homeostasis. Moreover, Jiang et al. described differences in *‘developmental*’ and *‘adult’* APCs, regulating adipose tissue development and maintenance, respectively (30). PDGFRα^+^ stromal cells are necessary for tissue formation and adipose tissue (AT) expansion during tissue development, whereas other APC populations contributed to adipocyte turnover and tissue homeostasis during adulthood (31). Additional studies identified further subtypes within the PDGFRα^+^ cell population, based on the effects of PDGFRα signaling to induce fibrosis (32). PDGFRα^+^ CD9^high^ cells were proliferative and induced fibrosis, whereas PDGFRα^+^ CD9^low^ cells showed an upregulation in pro-adipogenic transcription factors (**Figure 1**). However, PDGFRα^+^ CD9^low^ cells almost completely disappeared with AT fibrosis upon HFD feeding (33). Recently, a highly proliferative dipeptidyl peptidase-4 (DPP4)^+^ APC population was identified that gives rise to highly adipogenic ICAM1^+^ preadipocytes as well as a CD142^+^ adipogenic cell population, which was further studied by DPP4-Cre mediated lineage tracing experiments (**Figure 1**) (26, 34). Moreover, various other cell surface molecules have been described to distinguish APC and preadipocyte populations and we recently showed that the amino acid transporter ASC-1 regulates the commitment of APCs to become either white or beige adipocytes (35, 36). These findings highlight the heterogeneity within adipose progenitors that gives rise to different subsets of committed preadipocytes in AT. It is important to understand the complexity and diversity that is created by these subpopulation of cells to understand the mechanisms underlying tissue homeostasis and expansion. Moreover, these cells also interact with and are regulated by other cell types, especially immune cells, resident in AT.

## Interaction of Preadipocytes With Other Stromal Cells

The nervous-endocrine-immune system is an integrated regulatory network playing an essential role in maintaining homeostasis of the organism, including the adipose tissue (37, 38). The vasculature and neurons supply the adipose tissue with essential inputs such as nutrients, growth factors and signals from the autonomic nervous system. Thus, it is not surprising that in addition to adipocytes also APCs and preadipocytes continuously interact with other stromal cell types to regulate their function. APCs interact with endothelial cells through PPARγ mediated expression of VEGF, inducing vascular sprouting (**Figure 1**). This explains the perivascular localization of APCs (39). APCs also express PDGFRβ regulating APC-vascular niche interactions. These interactions are reciprocal as APCs are essential for vascular expansion, while vascular sprouting is essential for APC maintenance and differentiation. Most importantly, however, these interactions are necessary for adipogenesis and AT expansion (39).

In addition to the systemic inputs, immune cells modulate adipocyte function, as well as preadipocyte proliferation and differentiation either directly or indirectly (40–43). Many studies described adverse effects of chronic inflammation during obesity associated adipose tissue expansion (44). However, local inflammation is also, to some extent, necessary for healthy adipose tissue expansion and the MHO phenotype. Inhibition of pro-inflammatory pathways impairs adipogenesis, leading to ectopic lipid accumulation and systemic glucose intolerance (45, 46). The underlying mechanisms are still incompletely understood, but immune cell mediated reconfiguration of the ECM is certainly important for adipose tissue expansion. Moreover, the integration of various hormones and cytokines, in addition to nutritional cues, regulate processes of progenitor quiescence, proliferation and differentiation. To this end, we discuss some of the key regulatory inputs of different immune cell types on preadipocyte proliferation, differentiation and lineage (white versus beige adipocyte) commitment.

### Macrophages

**Figure 2:**
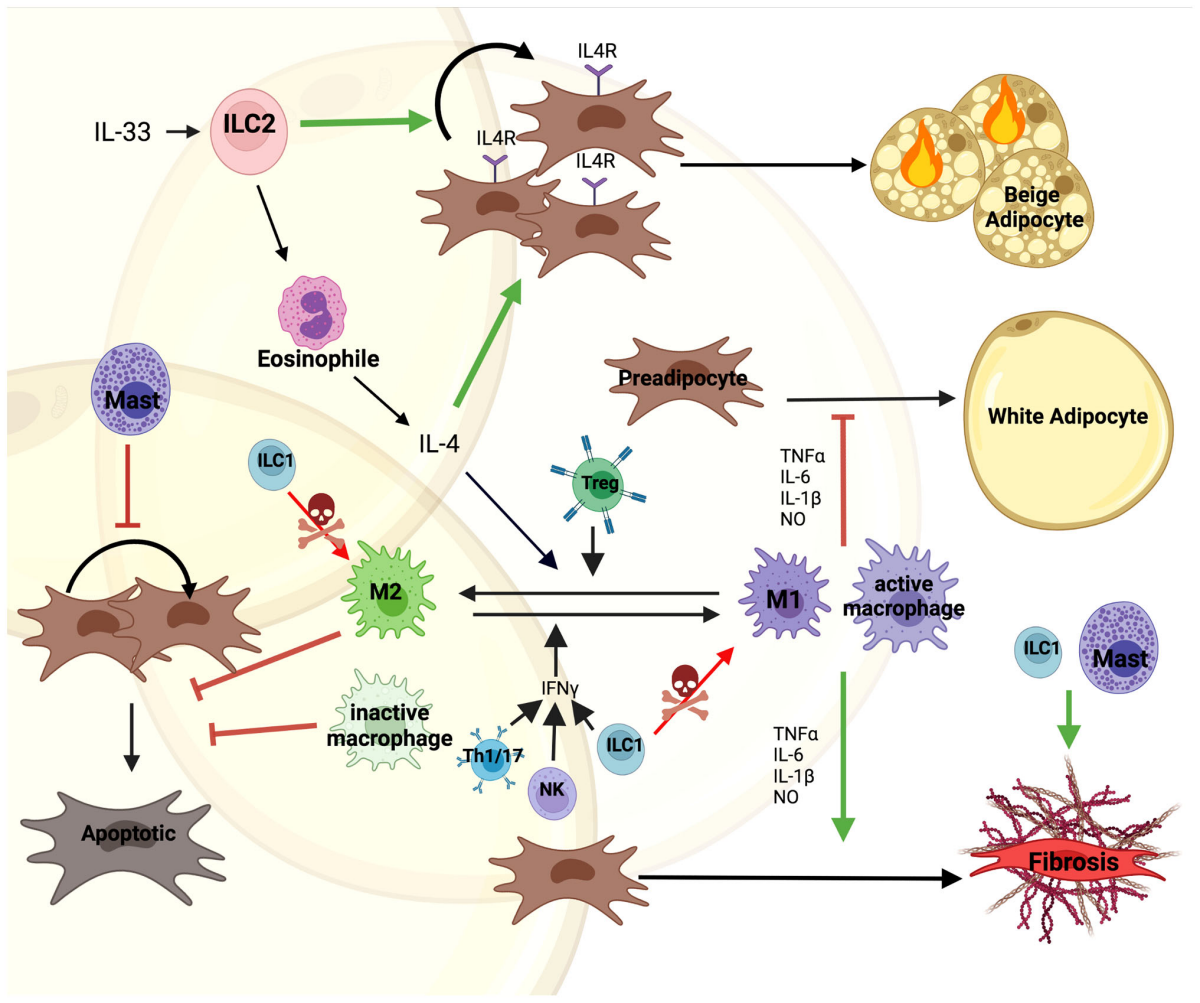
Direct and indirect interactions of immune cell with preadipocytes. Immune cells are one of the most diverse cell types constituting the microenvironment of AT. IL-33 mediated Type 2 Innate Lymphoid Cell (ILC2) activation induces secretion of IL-4 by eosinophils. This promotes both proliferation of IL4R expressing preadipocytes and their differentiation into beige adipocytes. Moreover, IL-4 along with Tregs promotes M1 to M2 macrophage polarization supporting M2 controlled survival of preadipocytes. On the other hand, Th1/17, Type 1 Innate Lymphoid Cells (ILC1) and Natural Killer (NK) cells secrete IFN-γ stimulating macrophage to polarize to the M1 phenotype, secreting pro-inflammatory factors, such as; TNFα, IL-6, IL-1β and nitric oxide (NO). This inhibits preadipocyte differentiation into white adipocytes and together with Mast cells induces a fibrotic phenotype. Mast cells also block proliferation of preadipocytes. Additionally, ILC1s kill macrophages and induce fibrosis in the tissue. The figure was generated using BioRender.com.

Macrophages are abundantly present and extensively studied in adipose tissue (47, 48). They play an important role in tissue homeostasis and hypertrophy induced adipose tissue inflammation and the initiation of local and systemic insulin resistance (49, 50). In general, accumulation of macrophages in adipose tissue is associated with insulin resistance and MUO (51). More recently, this was further refined as specific macrophage subtypes in visceral adipose tissue were found to be increased in diabetic obese compared to non-diabetic obese subjects (52). Previously, based on *in vitro* data, macrophages in adipose tissue were categorized as either M1 or M2. M1 macrophages originate from the stimulation with lipopolysaccharide (LPS), interferon (IFN)-γ or TNF-α, mediating pro-inflammatory actions by producing TNF-α, IL-6, IL-1β and nitric oxide (NO). M2 macrophages originate from the stimulation with IL-4, IL-10, IL-13, showing an anti-inflammatory phenotype by producing IL-4 and IL-10 (**Figure 2**) (53, 54). However, albeit widely used, the M1/M2 classification was recently challenged (55). Thus, previous *in vitro* data might not directly translate to the role of macrophages in adipose tissue *in vivo*. scRNAseq analysis of human white adipose tissues identified resident macrophages with an M1/M2-like phenotype. In contrast to the classical description of macrophages in *in vitro* studies, these subtypes were referred to as perivascular, inflammatory and lipid-associated macrophages. However, the interaction of these macrophage subtypes on progenitor cell fate has not been studied (47, 48, 52). Nevertheless, macrophages regulate tissue function through modulating preadipocyte expansion and commitment.

Relatively little is known on the role of macrophages and their secretome in regulating preadipocyte proliferation. Lacasa et al. used macrophage conditioned medium derived from *in vitro* differentiated blood monocytes of overweight patients. They showed increased proliferation but reduced differentiation of preadipocytes from non-obese donors when treated with the conditioned medium (56). Conversely, another human study found reduced preadipocyte proliferation when preadipocytes were exposed to macrophage conditioned media, which could be improved by antioxidant treatment (57). The discrepancy could be based on the different source of macrophages, as well as the physiologic and genetic background of the donors. A more detailed analysis of the impact of individual macrophage subtypes on preadipocytes is required to uncover the context specific role of macrophages on preadipocyte fate in humans.

Proliferation and differentiation are tightly linked with each other, as cell cycle exit is a prerequisite for adipocyte differentiation. Human and murine studies showed that M1 macrophages, the pro-inflammatory or classically activated macrophages, secrete factors inhibiting preadipocyte differentiation (56, 58, 59). The inhibitory effect was further confirmed by culturing CD14^+^ macrophages with preadipocytes of obese individuals, mediated by IKKβ/NF-κB signaling (60, 61). The block in differentiation was due to an impairment in the clonal expansion phase by inhibiting expression of cell cycle proteins such as; cyclin A, cyclin-dependent kinase 2, retinoblastoma protein, etc. (62, 63). Moreover, M1, pro-inflammatory macrophages promote preadipocytes to acquire a fibrotic phenotype, which is mediated through cytokines such as; TNF-α, IL-6 and IL-1β (**Figure 2**). This contributes to increased inflammation and tissue fibrosis impairing adipogenesis in both human and mice (56, 61, 64–66). However, these negative effects on preadipocyte differentiation were improved by growth hormone down-regulating IL-1β in macrophages (67).

Jang et al. compared high fat diet fed wild type to iNOS knockout mice and demonstrated that M1 macrophages produce nitric oxide impairing mitochondrial function by suppressing PGC1α expression. This inhibited adipocyte differentiation and promoted conversion of preadipocytes to a fibrotic phenotype (68). Furthermore, macrophages secrete the apoptosis inhibitor of macrophage, which also inhibited adipogenesis (69). In addition to proliferation and differentiation, maintenance and survival of existing preadipocytes is also controlled by macrophages, as macrophage conditioned medium promoted preadipocyte survival in a PDGF-dependent manner, which strongly stimulates PI3K-Akt and MEK-ERK1/2 pathways (70, 71). This effect was observed in M2 macrophages, activated by IL-4, but not activated M1 macrophages (72). Thus, the activation state of macrophages can have profound effects on preadipocyte survival, proliferation and differentiation and thereby shape the ability of the organism to store surplus energy in the future.

### Mast Cells

Mast cells (MCs) are tissue resident cells of the innate immune system that accumulate in adipose tissue with obesity (73). As previously reviewed, MC recruitment and activation has pleiotropic effects on adipose tissue (74). Importantly, MC-released IFN-γ, chymase, tryptase, IL-6, and cysteinyl cathepsins promote adipogenesis as well as angiogenesis through direct actions on adipocytes and endothelial cells as well as macrophages (52, 75, 76). Moreover, MCs play an important role in the reconfiguration of the extracellular matrix. Thus, MCs are a highly dynamic and important part of the nervous-endocrine-immune system regulating adipose function. Current data on the role of mast cells on preadipocyte differentiation are restricted to murine studies and controversial. Hirai et al. showed that mast cell protease 6 (MCP-6) secreted by mature mast cells induces collagen V expression in obese adipose tissue, contributing to adipose tissue fibrosis inhibiting preadipocyte differentiation (**Figure 2**) (77). Mast cell deficient Kit^w-sh/w-sh^ mice, which are fertile and non-anemic but also lack additional cell types mainly, melanocytes, and interstitial cells of Cajal, confirmed these previous findings and showed upregulated adipogenic capacity and preadipocyte proliferation (78). Similarly, mice where MCs were chemically inhibited as well as Kit^w-sh/w-sh^ mice showed an increase in sWAT PDGFRα^+^ APC proliferation and induced beige (thermogenic) adipocyte differentiation (79). However, stimulation of MCs by calcium or high-glucose induced adipogenesis in 3T3-L1 preadipocytes through the production of 15-deoxy-delta-12, 14-prostaglandin J2 acting as an activator of PPARγ (80). Furthermore, Chen et al. demonstrated that mast cell derived heparin could act as the endogenous factor initiating fascial adipogenesis (81). These findings suggest an adipose depot specific effect of mast cells on adipocyte progenitors, dependent on other immune cells and the metabolic state of the organism. Moreover, available data on the role of MCs on adipocyte progenitors focus on studies in rodents, while the role in humans remains to be determined.

### Innate Lymphoid Cells

Type 2 innate lymphoid cells (ILC2), which are part of the first line of the innate immune system, regulate type 2 immunity during tissue damage, parasite infection, and allergy (82–87). Apart from their role in wound healing, ILC2s also exhibit positive effects on metabolic homeostasis in visceral WAT, where they regulate eosinophils and alternatively activate macrophages (88). IL-33 mediated activation of ILC2s promote proliferation and commitment of APCs to become beige rather than white adipocytes (**Figure 2**) (89). In addition, an alternative pathway was described through the interaction of ILC2s with eosinophils, promoting secretion of IL-4, which activated IL-4R signaling in preadipocytes (89, 90). In the steady state, lacking IL-33, co-culture of adipose-derived ILC2s with 3T3-L1 cells induced adipocyte differentiation and lipid accumulation (91). Moreover, apart from ILC2s, ILC1s as well as NK cells play an important indirect role in modulating adipocyte-immune cell interactions. ILC1s kill adipose tissue macrophages and maintain macrophage homeostasis (**Figure 2**). However, this effect is abolished in obesity (92). Furthermore, adipose ILC1 secreted IFN-γ contributes to M1 macrophage polarization under HFD feeding (93, 94). Similar effects are also seen by NK cell activation (95, 96). Co-culture of ILC1 from obese subjects with lean stromal vascular cells upregulated the expression of fibrosis-related genes and ECM regulators, indicating that ILC1s promote fibrosis in obese adipose tissue (93).

### T Cells

The direct effects of T cells on preadipocytes is unclear. Pan et al. showed that senescent T cells inhibit brown preadipocyte differentiation by secreting high levels of IFN-γ (97). Co-culture of active pan T cells with 3T3-L1 preadipocytes *in vitro* significantly suppressed differentiation (98). However, individual T cell subtypes play distinct roles in regulating inflammation in adipose tissue by modulating the switch between macrophage phenotypes. Pro-inflammatory T cells, such as; Th1 and Th17, activate pro-inflammatory macrophages by secreting IFNγ and IL-17. However, anti-inflammatory T cells, like Th2 and Foxp3^+^ Treg induced macrophage differentiation into the anti-inflammatory macrophages by secreting IL-4 and IL-13 (**Figure 2**) (99). Interestingly, the percentage of naive CD4^+^ and CD8^+^ T cells were significantly higher in insulin sensitive compared to insulin resistant obese individuals, whereas activated T cells and IL-6 levels were reduced (100). This suggests that T cells contribute to the low-grade inflammation observed in MUO, albeit the link to alterations in adipose tissue has to be made.

Here we provide an overview of selected adipose tissue resident immune cells and their role in modulating preadipocyte fate directly or indirectly (**Figure 2**). Due to the complex regulations between immune cells and with the various adipocyte precursor populations, the impact of these interactions on the MHO phenotype remains to be determined. Moreover, adipose tissue depot specific differences in progenitors and immune cell composition need to be considered to evaluate the role of individual immune cell populations on healthy AT expansion. Furthermore, it will be exciting to see additional *in vivo* data on the regulations and interdependence of progenitor cell proliferation and differentiation on each other.

## Conclusion and Future Perspective

There is a rapidly increasing number of studies investigating adipocyte-preadipocyte-immune cell interaction. Thus, more and more details on the cellular subtypes and their regulation in obesity and the metabolic syndrome become available. Various computational approaches based on large–omics datasets are and will provide exciting new insights into the complex multidimensional spatial and temporal interactions of cell types, endocrine and nutritional inputs (47). This will allow a more holistic view on adipose tissues and their cellular interactions. However, these analyses are limited by the quality of the input data. Irrespective of the technical aspects of data acquisition, several points will need to be considered/improved to significantly advance our understanding of the control of adipocyte progenitor fate. One of the biggest limitations is the use of *in vitro* systems. Although simplification is important to identify molecular mechanism, the use of poorly defined progenitor or immune cell populations often results in data that cannot be translated to *in vivo*. Moreover, 2D cell culture models, most often do not allow “natural” cell-cell interactions, disregarding the effects of the ECM and biophysical properties such as oxygenation etc. Conversely, studying these mechanisms *in vivo* is also complex due to the lack of specific genetic models and low sample sizes. Moreover, the use of different mouse strains, in different life science disciplines, such as immunology and metabolism research also complicate the comparison of research data. To this end, novel developments such as spatial transcriptomics and single cell proteomics, could provide elegant ways to establish cell-cell interactions *in vivo*, most importantly in humans. Once these technical difficulties are solved, it will be fascinating to elucidate the reciprocal interaction of obesity and the metabolic syndrome with adipose stromal cell composition, function and activity. In summary, the expansion of adipose tissue, as well as the metabolic consequences of it in the context of MHO and MUO rely on a complex interplay between all cellular components, which should be studied as networks rather than linear interactions.

## Author Contributions

All authors listed have made a substantial, direct, and intellectual contribution to the work, and approved it for publication.

## Funding

XY received support from China Scholarship Council (No. 201908370218).

## Conflict of Interest

The authors declare that the research was conducted in the absence of any commercial or financial relationships that could be construed as a potential conflict of interest.

## Publisher’s Note

All claims expressed in this article are solely those of the authors and do not necessarily represent those of their affiliated organizations, or those of the publisher, the editors and the reviewers. Any product that may be evaluated in this article, or claim that may be made by its manufacturer, is not guaranteed or endorsed by the publisher.
